# Cost analysis and exploratory cost-effectiveness of youth-friendly sexual and reproductive health services in the Republic of Moldova

**DOI:** 10.1186/1472-6963-14-316

**Published:** 2014-07-21

**Authors:** Jari Kempers, Evert Ketting, Galina Lesco

**Affiliations:** 1 Qalys Health Economics, Middenweg 239 I, 1098AP Amsterdam, The Netherlands; 2 Nijmegen International Centre for Health Systems Research and Education (NICHE), Department of Primary and Community Care, Radboud University Nijmegen Medical Centre, PO Box 9101, 6500HB Nijmegen, The Netherlands; 3 Youth-Friendly Health Centre Neovita, Health for Youth Association, 19, Socoleni Str., MD 2020 Chisinau, The Republic of Moldova

**Keywords:** Adolescent, Youth-friendly, Centre, Sexual, Reproductive health, Prevention, Cost, Scale-up, Cost-effectiveness

## Abstract

**Background:**

Youth-friendly sexual and reproductive health services (YFHS) have high priority in many countries. Yet, little is known about the cost and cost-effectiveness of good quality YFHS in resource limited settings. This paper analyses retrospectively costs and potential cost-effectiveness of four well performing youth-friendly health centres (YFHC) in Moldova. This study assesses: (1) what were the costs of YFHSs at centre level, (2) how much would scaling-up to a national good quality YFHS programme cost, and (3) was the programme potentially cost-effective?

**Methods:**

Four well performing YFHCs were selected for the study. YFHS costs were analysed per centre, funding source, service and person reached. The costing results were extrapolated to estimate cost of a good quality national YFHS programme in Moldova. A threshold analysis was carried out to estimate the required impact level for the YFHSs to break-even (become cost saving).

**Results:**

Average annual cost of a well performing YFHC was USD 26,000 in 2011. 58% was financed by the National Health Insurance Company and the rest by external donors (42%). Personnel salaries were the largest expense category (47%). The annual implementation costs of a good quality YFHSs in all 38 YFHCs of Moldova were estimated to be USD 1.0 million. The results of the threshold analysis indicate that the annual break-even impact points in a YFHC for: 1) STI services would be >364 averted STIs, 2) early pregnancy and contraceptive services >178 averted unwanted pregnancies, and 3) HIV services only >0.65 averted new HIV infections.

**Conclusions:**

The costing results highlight the following: 1) significant additional resources would be required for implementation of a good quality national YFHS programme, 2) the four well performing YFHCs rely heavily on external funding (42%), 3) which raises questions about financial sustainability of the programme. At the same time results of the threshold analysis are encouraging. The result suggest that, together the three SRH components (STI, early pregnancy and contraception, and HIV) are potentially cost saving. High cost savings resulting from averted lifetime treatment cost of HIV infected persons are likely to off-set the costs of STIs and unwanted pregnancies.

## Background

Youth-friendly sexual and reproductive health services (YFHS) have high priority in many countries. Yet, little is known about the actual cost and potential cost-effectiveness of good quality YFHS in resource limited settings. This poses challenges for policy making, and for lobbying and fundraising for the YFHS programmes as well.

This study assesses retrospectively costs and potential cost-effectiveness of four well performing youth-friendly health centres (YFHC) in the Republic of Moldova. This paper provides new information that policy makers and programme managers can use to support planning, budgeting and implementation of similar YFHS programmes in other countries.

The purpose of this study was to determine: 1) How much did the YFHS programme cost in the four well performing YFHCs in 2011? 2) How were the YFHCs financed? 3) On what the YFHCs spent their funds? 4) How much did delivering each service cost? 5) How much did reaching one person cost? 6) How much would scaling up to a good quality national YFHS programme cost? and 7) Were the YFHSs cost-effective?

Young people aged 10–24 years represent a large share (22%) of the total population of Moldova [1]. Even though some adolescent sexual and reproductive health (SRH) indicators have improved during the last decade [2,3], young people in Moldova continue to face various challenges related to their SRH, i.e. STIs and HIV, unwanted pregnancies and abortions.

The government of Moldova has given high priority to the health and development of young people [4]. The Ministry of Health of the Republic of Moldova’s (MoH) efforts to develop a YFHS programme were supported by several international agencies including; the United Nations Children's Fund (UNICEF), the International Development Association (IDA), the United Nations Population Fund (UNFPA), the World Bank, and the Swiss Agency for Development and Cooperation (SDC) and the World Health Organization (WHO).

The establishment of the YFHS programme was a gradual process, which began in 2002 by creation of three pilot centres. This was followed by capacity building and founding the first ten YFHCs in 2005. In 2007 these centres were integrated in the state healthcare system as parts of primary healthcare centres. In 2011 the MoH initiated a process to scale-up YFHSs to the national level. As a result in January 2013, 38 primary healthcare centres provided YFHSs and reached approximately 70,000 young people annually [5].

In 2008 a major success was that the National Health Insurance Company (NHIC) started to support the YFHCs financially. At the time of writing this article (October 2013) NHIC was the largest financier of the YFHSs programme and all the 38 YFHCs were contracted by NHIC. However, the imbursements from NHIC are not sufficient to cover all operation costs of YFHCs. The NHIC financing is mainly focused on healthcare services and consequently many prevention, outreach, informational and educational activities remain under-funded.

The YFHCs provide integrated services for adolescents by using multidisciplinary teams. The YFHSs include: 1) sexual and reproductive health (SRH) services, 2) general health services, 3) psychological counselling, 4) information, education and communication (IEC) activities, 5) referrals, and 6) outreach work.

## Methods

This article is divided into two parts: cost analysis and threshold analysis.

### Selection of youth-friendly health centres

The objective was to evaluate costs of good quality YFHSs. The provided services and their quality vary between YFHCs. Therefore four well performing centres were selected out of 38 YFHCs. In this way the cost analysis reflects resources needed for implementation of a good quality YFHSs. The selection was based on the following criteria; 1) the centre has been operational for longer than three years, 2) it provides an extended YFHS package [6], 3) it meets the quality standards of YFHS [6], and 4) it obtained a high score in a baseline evaluation of YFHS Quality Standards in 2009 [7]. Moreover, to ensure that the sample is representative for the programme setting, two urban and two rural centres were selected. Table 1 summarizes the characteristic of the selected centres.

**Table 1:** Characteristics and catchment populations of selected four well performing YFHC in 2011

**Name of youth-friendly health centre (YFHC)**	**Location**	**Catchment population of YFHC(10–24 years)**	**Urban/Rural**
Neovita - the national centre	Chisinău - the capital and the largest city	^†^35,000	Urban
Atis	Bălți - third largest city	^††^27,000	Urban
Salve	Edinet - smaller town in the North district with many villages in the catchment area	^††^16,000	Rural
Tineri Pentru Tineri	Cimislia - smaller town in the South district with many villages in the catchment area	^††^15,000	Rural

^†^Estimated coverage 20% of age group 10 – 24 years of Chisinău [2]. ^††^Estimated coverage 50% of age group 10 – 24 years of city or town [2].

### Cost analysis

The following methods were used to answer the research questions on cost of the YFHS programme. The costs were analysed from a programme perspective (healthcare provider’s perspective). All the costs related to delivering the YFHS programme in the selected four centres were included in the analyses. Financial and material support from local authorities and international donors were included. Unpaid voluntary work and beneficiaries’ out-of-pocket payments (e.g. medicine costs in a pharmacy) were excluded. The time horizon of the cost analysis was one year. The cost analyses were conducted for year 2011, which was the closest complete financial year at the time of conducting this study. All costs are at 2011 prices and presented in 2011 USD. Costs in Moldovan leu (MDL) were translated to USD at a rate of 11.72 [8].

The cost analyses were based on financial records of: 1) Finance Services of Public Medical Health Institutions for YFHCs (FSPMHI), 2) NHIC, 3) National Centre of Reproductive Health and Medical Genetics (NCRHMG), 5) Health for Youth Association, 5) Family Doctors Centres (FDCs) and local authorities, and 6) information received from UNICEF, SDC and other donors.

Personnel salaries constitute a large part of the budget of the YFHS programme. A special time use form was developed and tested to measure how the personnel spend their working time at the centres. The self-reported survey was used to monitor time use of 28 salaried employees during a period of two weeks in September 2012. The collected data was statistically analysed by using SPSS software.

First, the cost of providing good quality services in the four YFHCs in 2011 were calculated. Then the total annual costs of the four YFHCs were analysed per financing source. Next, the total costs were presented in five standardized expense categories: 1) salaries, 2) medical supplies, 3) information materials, 4) personnel training, and 5) operations. *Salary costs* relate to gross salaries of the personnel of the YFHCs and the programme-related portion of salaries of financial and monitoring and evaluation personnel. *Medical supplies* include costs of: tests (smears, pregnancy and HIV tests), medicines for emergency care and medical materials used in the centres, distributed condoms and contraceptives. *Information materials* covers procurement and production costs of informational materials, brochures and leaflets. *Training costs* relate to training and capacity building of personnel of the four centres. *Operation costs* include computers, office supplies and -furniture, facilities and maintenance of the centres and transport costs.

The services of the YCHCs were grouped into six main categories. SRH services were: 1) STI, 2) HIV, and 3) early pregnancy and contraception. Other non-SRH services were: 4) general health services and 5) psychological counselling. In addition, informational and educational services were categorized as 6) IEC activities.

*STI services* include testing, diagnostics, treatment and follow-up consultations. *HIV services* include IEC HIV prevention activities, voluntary confidential counselling and testing (VCCT) at the centres or referrals to specialized service providers and in case of an HIV + result referral for treatment and social support. *Early pregnancy and contraceptive* services were grouped together. These cover information, contraceptive counselling and contraceptives distribution (condoms, COCs); pregnancy diagnostic (tests, examination, USG), referral for safe abortion or antenatal care, and social support. Currently only Neovita centre has permission to offer safe abortion services; for other centres abortions are conducted elsewhere. *General health services* include consultations related to a variety of medical advices and interventions, as well as health promotion activities. *Psychological counselling* include psycho-emotional, violence related and substance abuse information, counselling and referral.

*‘Costs per service type’* were calculated as follows: 1) the salary costs were divided into: medical service-, IEC activity- and overhead salaries. The distribution was based on the results of the time use survey. 2) The medical service and IEC salary costs were allocated to each service type according to percentage of used working hours. 3) The remaining overhead salary costs were divided between medical services and IEC activities according to the number of working hours. 4) Personnel training costs were handled in the same way as the salary costs. 5) All medical supply costs were allocated to medical services. Additional costs for rapid HIV tests were added for HIV services, because YFHCs are planning to start providing rapid VCCT services in the near future. 6) Information material and operation costs were split between medical services and IEC by using number of services delivered in 2011. Finally, the cost per service was calculated by dividing the allocated budget by the number of services delivered.

In order to estimate *‘cost per person reached’*, the services were divided into two groups: 1) Healthcare services, and 2) IEC activities. Next, the following assumptions were made: for all the healthcare services there were on average two consultations per patient, and for IEC services there was one activity per person. These assumptions were based on expert opinions of employees of the YFHS programme.

Finally, national level cost of scale-up good quality YFHSs to all 38 YFHCs of Moldova were estimated, by extrapolating average budget of three smaller well performing centres: Atis, Salve and Tineri Pentru Tineri. The Neovita centre was excluded from the extrapolation, because it fulfils several national functions for the programme and has a much higher budget than other centres. The purpose of this extrapolation was to provide MoH with an estimation for national level decision making on funds needed on scale-up of good quality YFHSs.

### Threshold analysis

The following methods were used to answer the research questions on potential cost-effectiveness of the YFHS programme. It was not possible to carry out a classical retrospective cost-effectiveness analysis, because this requires: 1) impact assessment of the YFHSs programme, and 2) comparison with the incidence of infections and unwanted pregnancies in a comparable area where the YFHS do not exist. Such data was not available. Therefore, cost-effectiveness was approached from a different angle. Threshold analyses were conducted on: *What would be the required impact levels for SRH services to break-even and become cost saving in a well performing centre in 2011?*

The threshold analysis focuses on three SRH outcomes: 1) STIs, 2) unwanted pregnancies, and 3) HIV infections. Other non-SRH services (general health services and psychological counselling) were excluded from the analysis. The cost of delivering the SRH services were compared with cost savings resulting from averted treatment costs. The averted treatment costs are cost savings resulting from avoided negative SRH outcomes. Calculations were made to estimate break-even points for each SRH outcome. A break-even point is a point where the cost of delivering a healthcare service and cost savings resulting from averted treatment costs are equal. An intervention is cost saving, if the number of averted cases is higher than at the break-even point. Please note that this is a hypothetical analysis on impact levels *required* for the SRH services to break-even, not an actual impact assessment of the programme.

The threshold analysis was carried out from a healthcare provider’s perspective. Costs related to: 1) delivering the SRH services (results of the costing part of this study) and 2) cost savings resulting from averted treatment costs, were included in the analyses.

In the threshold analysis different STIs were grouped as a ‘generic STI’. This was done because in the YFHS programme costing the expenses were analysed on healthcare service group level (in this case all STI services), not per treated infection. The STI costs included testing, diagnostics and follow-up consultations, and cost of IEC services related to STI prevention as well. A typical STIs treatment consist of three consultations and cost USD 15.06 in the YFHCs. Treatment costs of syphilis and gonorrhoea were covered by the NHIC and were therefore included in the analysis (USD 220) [9]. According to medical personnel of the YFHCs 5% of STI patients have syphilis or gonorrhoea. Patient’s out-of-pocket medicine costs were excluded from the analysis. The time horizon for the treatment cost was limited to successful treatment of an STI. The following parameters were used to estimate cost savings resulting from treatment of STIs in the YFHCs: 1) Moldovan adolescents, who have casual sexual partners (i.e. 38% of all sexually active adolescents) [10], have on average 3.5 casual sex partners per year [10], 2) condoms are used in 52.8% of these intercourses [10], and 3) an STI infected adolescent would infect on average 1.9 partners in a year (calculations based points 1 and 2).

Early pregnancy and contraceptive services were categorized as ‘unwanted pregnancy’ related. The consequences of an unwanted pregnancy were divided into: 1) abortions (34%), and 2) deliveries (66%) [11]. The time horizons for the costs were limited to completion of an abortion or a delivery. Abortion costs were limited to medicine costs of medical abortions (USD 38) [12]. According to medical personnel of Neovita YFHCs, 95% of the girl patients, who decide to have an abortion, choose for a medical abortion. According to Boderscova (2005) 45% of deliveries in the age group 15–19 years in Moldova were normal deliveries and 55% had moderate or severe complications [13]. Delivery costs include: 1) normal delivery USD 141 [9], 2) delivery with complications USD 327 [9], 3) ANC co-payment (USD 30) [9] for all deliveries. Importantly, not all teenage births are unwanted. Results of the latest available Demographic and Health Survey in Moldova 2005 indicate that the weighted average (recalculated) percentage of unwanted births among all births to mothers up to 24 years old was 19.2% [14].

‘HIV services’ included general IEC HIV prevention activities and VCCT. UNAIDS report on Assessment of Expenses for Antiretroviral Therapy for People Living with HIV and AIDS [15] was used as a source for lifetime treatment cost of patients on first-line of ARV in Moldova. The expected remaining lifetime of an HIV infected person was assumed to be 21.5 years [16]. The lifetime treatment costs were discounted with 3.5% annually to present the value of costs, as recommend by the National Institute for Health and Clinical Excellence [17].

## Results

### Cost analyses

This section answers the research questions on cost of the YFHS. Please note that, unless otherwise stated, all the results of cost analyses represent the selected four well performing YFHCs, not the entire YFHS programme.

*How much did the YFHS programme cost in the four well performing YFHCs in 2011?* The total annual costs of delivering good quality YFHSs in the selected four YFHCs were USD 164,000 in 2011. Neovita, the largest centre, had annual cost of USD 89,000. Costs of Atis centre were USD 32,000. This was followed by the two smaller rural centres; Salve USD 24,000 and Tineri Pentru Tineri USD 20,000.

**Figure 1:**
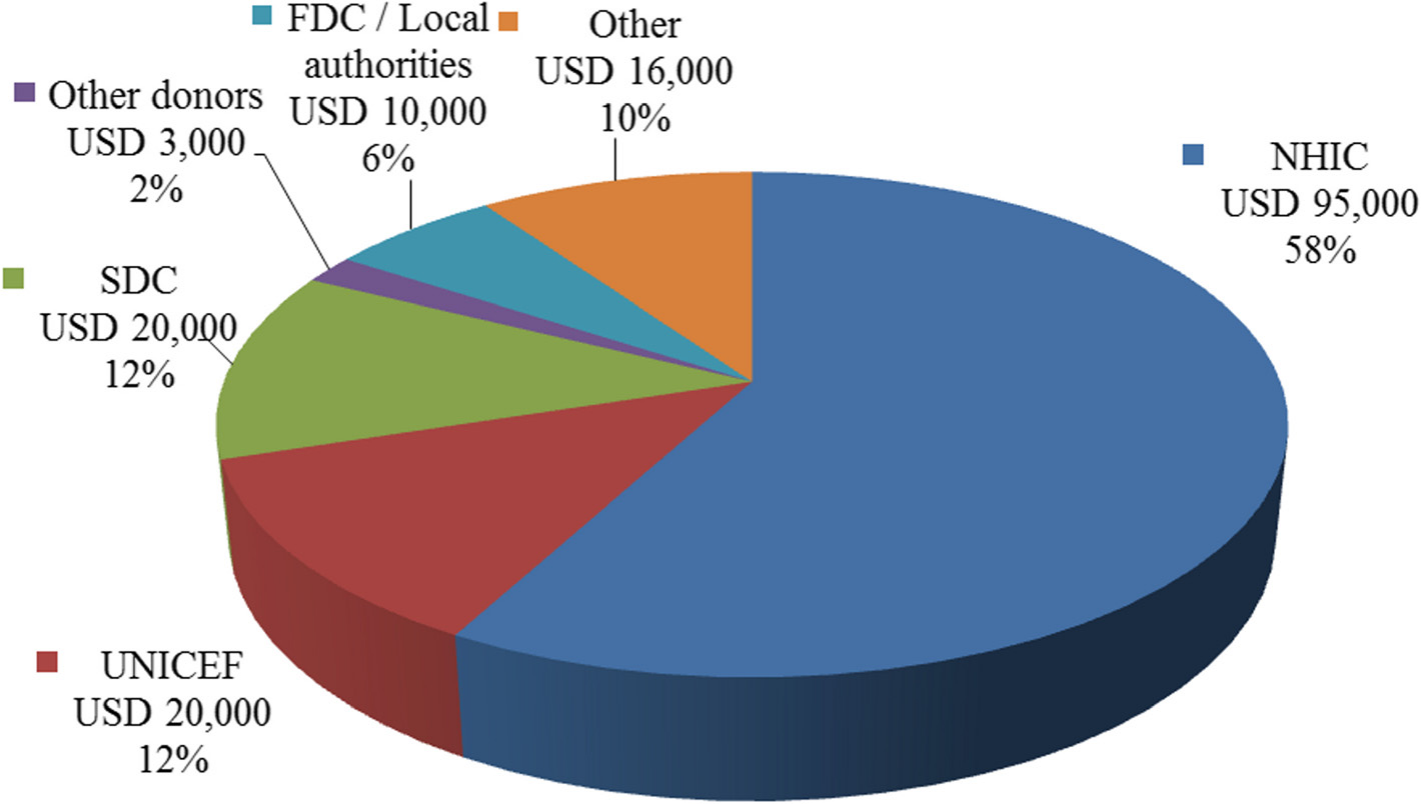
Financing sources of the four YFHCs in 2011.

*How were the four YFHCs financed in 2011?* Figure 1 provides an overview of financing sources of the four YFHCs in 2011. The NHIC was the largest financier, covering 58% of the costs (USD 95,000). Support from UNICEF was 12% (USD 20,000) and from Swiss Development Cooperation (SDC) 12% (USD 20,000). Other donors accounted together for 2% (USD 3,000). Contributions from FDCs and local authorities were 6% (USD 10,000) and other sources 9% (USD 16,000).

**Figure 2:**
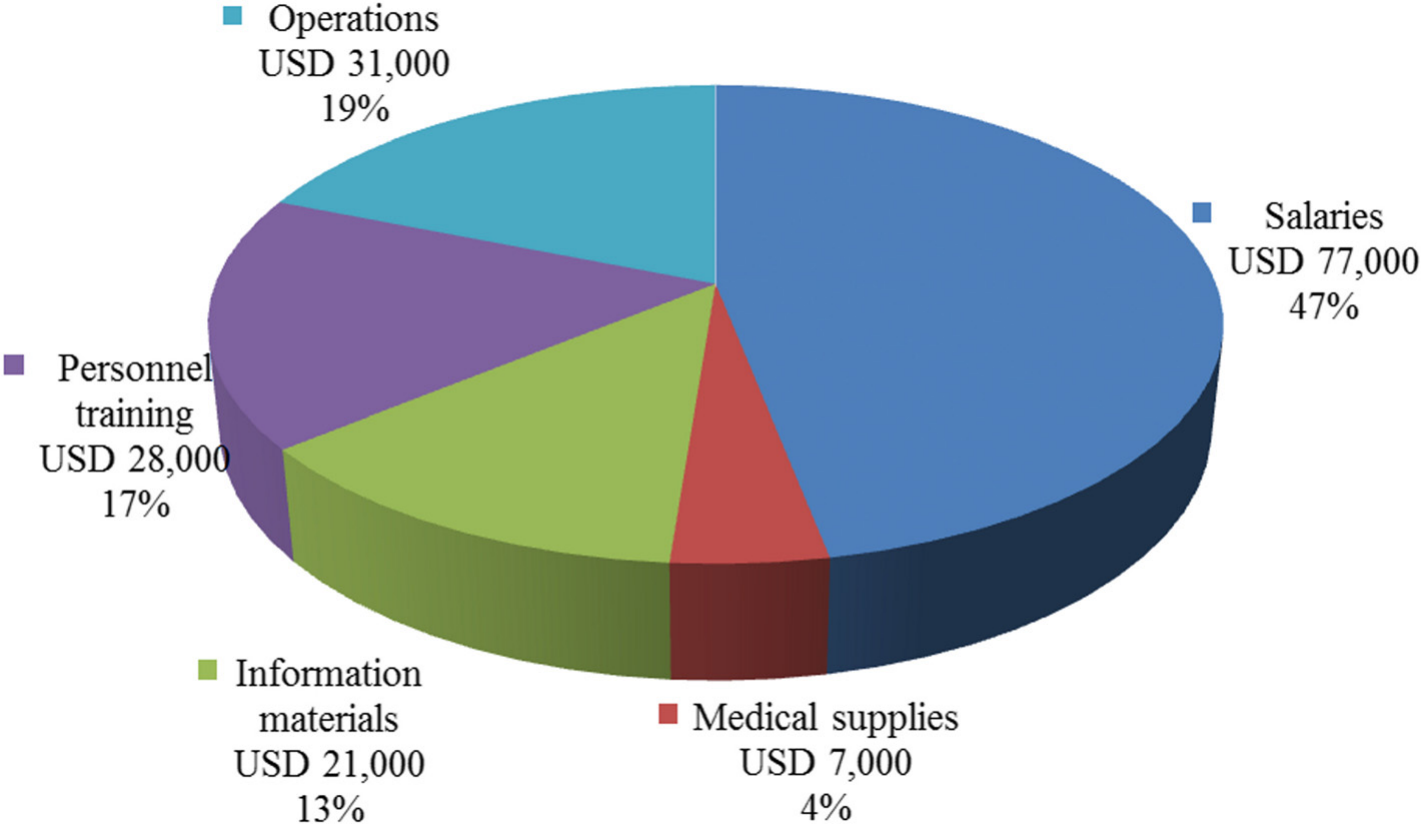
Breakdown of total cost of the four YFHCs in 2011.

*On what the four YFHCs spent their funds in 2011?* A breakdown of total costs of the four YFHCs in 2011 is shown in Figure 2. Personnel salary costs were by far the largest cost category, with 47% (USD 77,000). The second largest group was operation costs: 19% (USD 31,000). Third were personnel training costs 17% (USD 28,000). Together the two personnel related categories; salaries and personnel training, cover 64% of the total costs. Information materials were the fourth cost category and accounted for 13% (USD 21,000). Medical supplies were the smallest group: 4% (USD 7,000).

*How much did delivering each service cost in 2011?* Table 2 summarizes the calculations of cost per healthcare service and IEC activity. The costs column shows the budget allocated for each service type. The services column indicates the number of services provided. The last column shows the average cost per service. An STI consultation cost USD 5.02. An HIV consultation was USD 10.08. Patient contacts related to early pregnancy and contraception were USD 5.11. Other SRH services were USD 3.96. Psychological counselling services were the most expensive at USD 10.59. This is due to longer duration of these consultations. General health service consultations were USD 5.40. IEC activities had the lowest cost at USD 2.55, because these services are provided mainly to groups. An average group size was 20 persons.

**Table 2:** Costs per service category, number of services and cost per service in 2011

**Healthcare service**	**Costs (USD)**	**Services**	**USD per service**
STI consultations	29,000	5,780	5.02
HIV consultations	26,000	2,580	10.08
Early pregnancy & contraception	23,000	4,500	5.11
Other SRH	11,000	2,780	3.96
Psychological counselling	18,000	1,700	10.59
General health consultations	17,000	3,150	5.40
IEC group activities	51,000	20,000	^†^2.55

^†^Cost per person reached, not cost per IEC group activity.

*How much did reaching one person cost in 2011?* The allocated budget for all the healthcare services provided by the four YFHCs was USD 124,000 in 2011. In total there were 20,490 healthcare consultations. It was assumed that on average there were two consultations per patient. Therefore, the cost of providing YFHS (healthcare services only) to one person cost USD 12.10. IEC activities’ budget was calculated to be USD 51,000. In total 20,000 persons were reached by these services in 2011. Therefore the average cost of providing IEC services to one person was USD 2.55. These calculations rely on assumptions on the budget allocations, number of services delivered and contacts per person. Therefore, the cost per person reached should be interpreted as estimations.

*How much a good quality national YFHS programme would cost?* Average annual budget of the three smaller centres; Atis, Salve and Tineri Pentru Tineri, was approximately USD 26,000 per YFHC. There are 37 YFHCs + the larger Neovita centre. If all the 38 centres would provide good quality YFHSs, the annual cost of a national programme would be USD 1,051,000 (Calculations: 37 centres * USD 26,000 + Neovita USD 89,000).

### Threshold analysis

This section answers to the research questions on potential cost-effectiveness of the YFHS. The results of threshold analyses represent one well performing YFHC, not for the entire YFHS programme. The threshold analysis measures break-even impact points for 1) STI, 2) pregnancy and contraceptive, and 3) HIV services. Please note that this is a hypothetical analysis on impact levels required for the SRH services to break-even, not an actual impact assessment.

**Figure 3:**
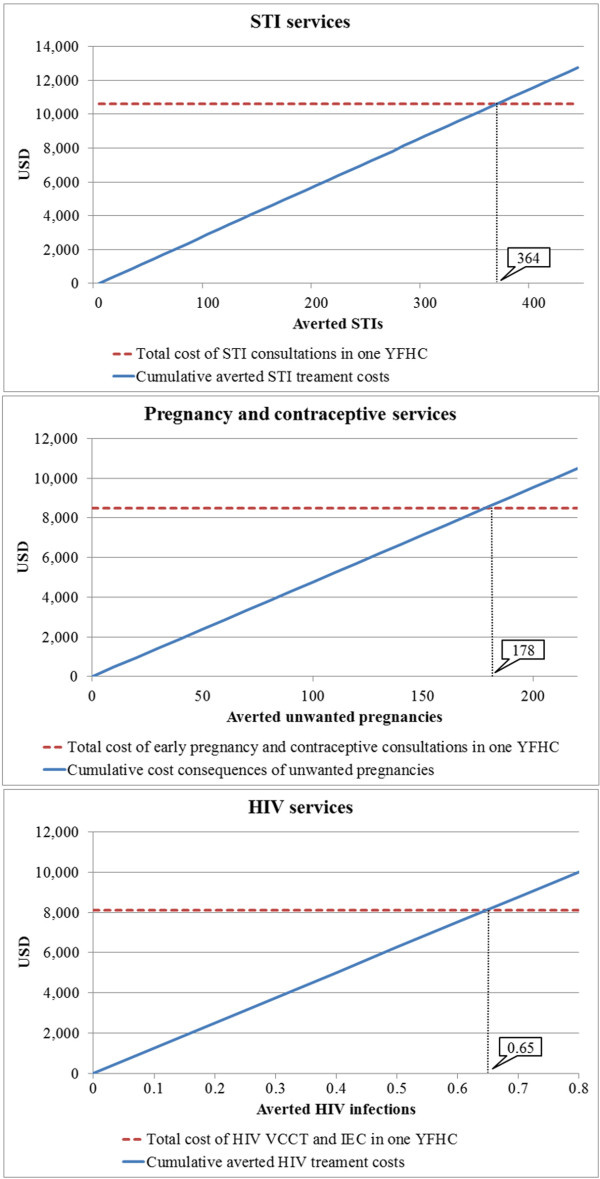
Break-even points for STI services, pregnancy and contraceptive services, and HIV services in a YFHC in 2011.

The total costs allocated to STI consultations and STI related IEC activities were on average USD 10,600 per a centre in 2011. The cost consequences of delayed treatment of STIs were estimated to be USD 29.1 (calculations based on 1.9 infected partners and 3 consultations per treated STI). Consequently the STI services would break-even if *364 STIs* would be averted as a result of the services in a YFHC in 2011. In other words, the STI services of a YFHC would be cost saving, if more than 364 STIs were averted.Figure 3 illustrates the break-even points for the STI services, pregnancy and contraceptive services, and HIV services. The horizontal dotted lines are the total cost of delivering the services in a YFHC in 2011. The horizontal axes are the number of averted cases (STIs, unwanted pregnancies or HIV infections) as a result of the services provided by the centre. The solid lines represent cumulative cost savings resulting from averted treatment costs. The points where the dotted and solid lines cross are the break-even points.

The average costs allocated to early pregnancy and contraceptive services were USD 8,500 per centre in 2011. The cost consequences of unwanted pregnancies (100% of abortions and 19.2% of deliveries) were estimated to be USD 47.8 (weighted average calculations based on costs of medical abortions and deliveries). The break-even point for the early pregnancy and contraceptive services would be *178 unwanted pregnancies* (ending in a medical abortion or an unintended birth), that would be averted as a result of the services in a centre in 2011 (Figure 3). The early pregnancy and contraceptive services would be cost saving, if more than 178 unwanted pregnancies were averted.

On average USD 8,100 was allocated to HIV components (IEC activities and VCCT) per a centre in 2011. Discounted (3.5%) lifetime treatment costs of HIV patients on first-line ART in Moldova were estimated to be USD 12,500 (undiscounted USD 17,000). Based on this, the HIV services would break-even if *0.65 HIV infections* would be averted as a result of the HIV services provided by a YFHC in 2011 (Figure 3). The HIV services would be cost saving, if more than 0.65 new HIV infections were averted.

Table 3 summarizes total cost of services, cost savings resulting from averted treatment cost and break-even points per SHR outcome.

**Table 3:** Total costs, cost savings resulting from averted treatment costs and break-even point per SRH outcome in a YFHC in 2011

**SRH outcome**	**Total costs (USD)**	**Cost savings (USD)**	Break-even point*
STI	10,600	^†^29.1	364
Unwanted pregnancy	8,500	^††^47.8	178
HIV	8,100	^†††^12,500	0.65

*Number of averted cases required to make the intervention cost saving in one YFHC in one year. ^†^Weighed cost of STI consultations, including syphilis or gonorrhoea treatment. Excluding patients’ out-of-pocket medicine costs. ^††^Weighed cost of medical abortions, normal deliveries, deliveries with complications and ANC co-payments. ^†††^Discounted lifetime treatment cost of an HIV + patient on first-line ART in Moldova, with expected remaining lifetime of 21.5 years.

## Discussion

### Cost analysis

The cost analysis of the four well performing YFHCs indicated that an average annual budget of a centre was approximately USD 26,000 in 2011. Neovita, the national centre, had a higher annual budget of USD 89,000. 58% of budgets of the four well performing YFHC were financed by NHIC and 42% by other financing sources (donors, FDCs and local authorities) in 2011. Personnel salary costs were the largest cost category (47%). Only 4% was used for medical supplies. If all the 38 YFHCs of Moldova would provide good quality YFHSs, the costs of a national YFHS programme were estimated to be USD 1,051,000.

The large share (42%) of external funding raises questions on financial sustainability of the programme. YFHSs are labour intensive services and therefore the share of personnel salary costs is high. Moreover, many activities, i.e. peer to peer education, are carried out by a large group of unsalaried volunteers. The volunteers play an import role in implementation of the programme and without their voluntary inputs the personnel salary costs could be significantly higher. Cost share of medical supplies was surprisingly low, especially when taking into account that this is a medical programme. There are two probable reasons for this: 1) beneficiaries pay for medicines by themselves, which may hinder vulnerable adolescents’ access to treatment, and 2) limited procurement of medical supplies due to financial constrains the YFHCs. Scale-up of good quality YFHSs to all 38 YFHCs of Moldova would require a significant funding increase from the NHIF and additional training and management support from MoH. At the time of writing this paper most of the newly established YFHCs were only starting to implement YFHSs and train their personnel. These centres did not yet provide all the planned YFHSs nor did they meet YFHS quality standards set by the MoH.

### Threshold analysis

The results of threshold analysis indicate that 364 STIs would need to be averted for the STI services to break-even and become cost saving in a YFHC. 178 unwanted pregnancies (100% of abortions and 19.2% of deliveries) would need to be averted in a year for the early pregnancy and contraceptive services to break-even in a YFHC. Only 0.65 new HIV infections would be need to be averted in a year for the HIV services to break-even in a YFHC.

The number of STIs required to be averted is relatively high (364 averted STIs), because STI treatments are typically low cost and the long-term cost consequences of non-treatment are limited. The break-even point for unwanted pregnancies is lower (178 averted unwanted pregnancies). This is driven down by the cost of carrying a pregnancy to term and delivery assistance, which are approximately five times higher than the cost of a medical abortion. Most importantly, very few HIV infections (0.65) would needed to be averted for the HIV services to break-even and become cost saving, because the long term cost consequences of new HIV infections are substantial. The lifetime treatment costs of HIV patients on first-line ART in Moldova were estimated to be USD 12,500 (discounted).

### Limitations

The analyses have some limitations. Firstly, personnel salaries are the largest part of the total budget. Therefore, the costing results are sensitive to variation in the number of employees and their salaries. Secondly, the cost analyses per service type and per person reached should be interpreted as estimations, because these calculations rely on assumptions on the budget allocations, number of services delivered and contacts per person. Third, the budget extrapolation for national scale-up does not take into account additional resources needed for coordination and training needed for a larger programme. Fourth, consultations included in the threshold analysis are often combinations of the SRH services (e.g. STI consultation and HIV testing). The results are influenced by how the costs are allocated to each SRH service group. Fifth, the largest cost savings are resulting from averted HIV infections. Therefore, the results are sensitive to variations of the HIV treatment costs and expected additional lifetime for patients with HIV. Finally, some consequences of unwanted pregnancies could not be measured in monetary terms.

## Conclusions

Cost analysis shows that significant additional resources would be required for implementation of a good quality national YFHS programme in Moldova. Approximately USD 1.0 million would be required annually to implement it in all 38 YFHCs. Moreover, the costing results highlight that the four well performing YFHCs rely heavily on external funding (42%) and on voluntary work, which raises questions about financial sustainability of the programme.

At the same time results of the threshold analysis are encouraging. The results suggest that, together the three SRH components (STIs, early pregnancies & contraceptives and HIV) are *potentially* cost saving. This is because of high cost savings resulting from averted HIV infections are likely to off-set the costs caused by STIs and unwanted pregnancies. SRH consultations are often a combination of services (e.g. STI consultation and HIV testing). Therefore, the results of threshold analysis should be interpreted as a whole.

The authors hope that the analysis of the economic aspects of the YFHS programme will contribute to the decision-making and implementation process in Moldova and in other countries planning to implement YFHS programmes.

## Competing interests

The authors declare that they have no competing interests.

## Authors’ contributions

JK is a health economist consultant (MSc in Health Economics). EK is a sexual and reproductive health & rights expert (PhD). GL is a medical doctor and head of Neovita YFHC in Chisinau and national coordinator of YFHS scale-up project in Moldova. JK and EK designed the study. JK and GL coordinated the data collection. JK performed the health economic analyses. GL and EK were responsible for the description and context of the YFHS programme. JK and EK wrote the manuscript. All authors read and approved the final manuscript.

## Pre-publication history

The pre-publication history for this paper can be accessed here:

http://www.biomedcentral.com/1472-6963/14/316/prepub
